# Protocol for the Foot in Juvenile Idiopathic Arthritis trial (FiJIA): a randomised controlled trial of an integrated foot care programme for foot problems in JIA

**DOI:** 10.1186/1757-1146-2-21

**Published:** 2009-06-30

**Authors:** Gordon J Hendry, Deborah E Turner, John McColl, Paula K Lorgelly, Roger D Sturrock, Gordon F Watt, Michael Browne, Janet Gardner-Medwin, Lorraine Friel, Jim Woodburn

**Affiliations:** 1 School of Health and Social Care, Glasgow Caledonian University, Cowcaddens Road, Glasgow, UK; 2 Department of Statistics, University of Glasgow, University Avenue, Glasgow, UK; 3 Division of Community Based Sciences, University of Glasgow, University Avenue, Glasgow, UK; 4 Centre for Rheumatic Diseases, University of Glasgow, University Avenue, Glasgow, UK; 5 Department of Child Health, University of Glasgow, University Avenue, Glasgow, UK; 6 NHS Greater Glasgow and Clyde, Glasgow Royal Infirmary, 16 Alexandra Parade, Glasgow, UK

## Abstract

**Background:**

Foot and ankle problems are a common but relatively neglected manifestation of juvenile idiopathic arthritis. Studies of medical and non-medical interventions have shown that clinical outcome measures can be improved. However existing data has been drawn from small non-randomised clinical studies of single interventions that appear to under-represent the adult population suffering from juvenile idiopathic arthritis. To date, no evidence of combined therapies or integrated care for juvenile idiopathic arthritis patients with foot and ankle problems exists.

**Methods/design:**

An exploratory phase II non-pharmacological randomised controlled trial where patients including young children, adolescents and adults with juvenile idiopathic arthritis and associated foot/ankle problems will be randomised to receive integrated podiatric care via a new foot care programme, or to receive standard podiatry care. Sixty patients (30 in each arm) including children, adolescents and adults diagnosed with juvenile idiopathic arthritis who satisfy the inclusion and exclusion criteria will be recruited from 2 outpatient centres of paediatric and adult rheumatology respectively. Participants will be randomised by process of minimisation using the Minim software package. The primary outcome measure is the foot related impairment measured by the Juvenile Arthritis Disability Index questionnaire's impairment domain at 6 and 12 months, with secondary outcomes including disease activity score, foot deformity score, active/limited foot joint counts, spatio-temporal and plantar-pressure gait parameters, health related quality of life and semi-quantitative ultrasonography score for inflammatory foot lesions. The new foot care programme will comprise rapid assessment and investigation, targeted treatment, with detailed outcome assessment and follow-up at minimum intervals of 3 months. Data will be collected at baseline, 6 months and 12 months from baseline. Intention to treat data analysis will be conducted.

A full health economic evaluation will be conducted alongside the trial and will evaluate the cost effectiveness of the intervention. This will consider the cost per improvement in Juvenile Arthritis Disability Index, and cost per quality adjusted life year gained. In addition, a discrete choice experiment will elicit willingness to pay values and a cost benefit analysis will also be undertaken.

**Trial Registration:**

Trial registration number: UKCRN5045

## Background

Juvenile Idiopathic Arthritis (JIA) is the commonest rheumatic disease in childhood with a variable worldwide prevalence ranging from 0.07 to 4.01 per 1000 children [1], while in the UK the prevalence is estimated at between 0.65 and 2.0 per 1000 children [1-3]. Foot problems have been reported as being common in JIA with 90% of children in a cross-sectional survey presenting with at least one foot problem associated with the disease process [4]. Foot and related problems attributable to JIA include synovitis, deformity, pain, stiffness, limited joint range of motion, enthesitis, and bony erosions [4-8]. As such, many sufferers have a reduced capacity to function [9] poorer health related quality of life (HRQOL) [10,11], and high health care costs have been reported [12,13].

Significant advances in the pharmacological management of JIA have taken place with the advocated earlier use of intra-articular cortico-steroid injections (ICIs), disease modifying anti-rheumatic drugs (DMARDs) and biologic therapies. These drugs have greatly improved the treatment options for paediatric rheumatologists, and functional outcomes appear to have improved as a result [14,15]. This shift towards earlier and more aggressive medical management has not been accompanied by specific foot related research. Further investigation is required to determine the prevalence and severity of foot problems in JIA, and also to evaluate the effectiveness of the current available treatments for such problems in the short and long term.

Our recent survey of foot problems and medical and podiatric management in a small 30 patient cohort with JIA found that patients with JIA can experience mild to moderate foot related impairments and disability despite medical management in line with modern day treatment paradigms [16]. Podiatry care included provision of customised functional foot orthoses, intrinsic muscle stretching and strengthening exercises, footwear advice and silicone digital splinting appliances. These podiatric treatment methods appeared to be in line with current recommendations [17]. However there are no recognised podiatry clinical practice guidelines for foot care in JIA and supporting evidence of efficacy is lacking. Foot orthoses have been evaluated in one small prospective study indicating short-term (3 month) reduction in pain and improved function and quality life with greater benefits from custom versus prefabricated devices or athletic shoes alone [18]. The study is limited in that the sample did not include adults with JIA, and only patients with foot or ankle involvement for a period of 2 years or less were recruited. This reduces the ability to generalise the results of the study to a wider population of patients with JIA who have foot involvement, and this is acknowledged by the authors in the discussion of study limitations [18].

Foot problems are often neglected as patients often reach podiatry too late for effective intervention, possibly due to technical difficulty or lack of recognition [19]. Our recent retrospective analysis of patient case-notes showed that only 18 of over 250 patients with JIA at one paediatric rheumatology centre were referred for podiatric opinion over a one year period [16]. However the authors acknowledge that some patients may have been referred to the orthotics service. To date there have been no other reports of the frequency of referral of patients with JIA from paediatric rheumatology to podiatry services. It appears probable that there is an unmet need for foot care within this population. Problems with delay in access to appropriate care in JIA are well recognised. There is a suggestion that there is a small therapeutic window of opportunity which if missed results in prolonged untreated active disease and poor long-term outcome [20].

Treatment for foot and ankle disease in JIA has focused on the use of intra-articular cortico-steroid injections (ICIs), physiotherapy, orthoses and orthopaedic surgery as an adjunct to medical care to both resolve synovitis and to correct or maintain foot posture and function. By adding podiatry to usual medical care, thus relying on largely palliative mechanical based treatments, it appears unlikely that a significant improvement in foot health is attainable. In order to provide optimum foot care, it is essential that disease activity and early joint destruction be monitored [21]. Use of a sensitive imaging technique can be adopted in patients with early arthritis to obtain accurate identification of the anatomical damage caused by inflammatory processes [22]. The current gold-standard in diagnostic imaging is magnetic resonance imaging (MRI). In comparison ultrasonography (US) is an inexpensive, readily accessible and valuable diagnostic imaging technique which can demonstrate both inflammatory and destructive changes [23-25] and improve the efficacy of ICIs [25,26].

In a clinical setting podiatric assessment of patients with JIA includes visual observation of gait, obtaining a detailed patient history, and physical examination. However simple visual observation of gait seems insufficient for identifying often subtle deviations from 'normal' gait patterns. Fairburn *et al *[27] demonstrated that instrumented gait analysis has a valuable role in JIA to provide a clear description between primary gait abnormalities and secondary compensations and to allow optimal targeting of physiotherapy and orthotic interventions. Therefore, by employing ultrasonography and instrumented gait analysis, both specific lesions (synovitis/tenosynovitis/enthesitis/erosions) and functional deficits (antalgia/asymmetry) may be detected and targeted with appropriate interventions such as ICIs, customised orthoses and physical therapies.

Medical treatments in JIA appear to be well driven by outcome as preliminary definitions for improvement, minimal disease activity, inactive disease and clinical remission have been developed using the core outcome variables. These provide physicians with realistic therapeutic objectives [28-30]. Without objective and specific measures of foot related impairments and disability it is unlikely that clinical improvements can be detected. Currently there are no such outcome measures used in the routine podiatric care of children with JIA despite the recent development and validation of a disease and foot specific outcome measure; the JAFI [31]. The administration of a simple clinical measure such as the JAFI questionnaire as an appendage to routine medical note taking could permit objective monitoring of patients over time. Furthermore, clinicians can use clinical outcome measures to set realistic therapeutic goals in order to gauge improvements in their patients' conditions following interventions.

To summarise, there is a paucity of evidence to support simple podiatry alone as a complex intervention for foot problems in JIA patients. However significant improvements in the delivery of overall foot care may lead to a substantial increase in the uptake of this service. Utilisation of standardised and validated outcomes, as well as employing imaging techniques and thorough gait analysis should allow accurate monitoring, evaluation and appropriate alteration of treatments to achieve maximum treatment efficacy.

The FiJIA (Foot in Juvenile Idiopathic Arthritis) group aim to conduct an exploratory non-pharmacological phase II [32] randomised controlled trial of a new podiatry led integrated foot care programme versus current standard care podiatry for JIA patients with foot problems. The primary aim of this study is to evaluate the clinical and cost effectiveness of standard care podiatry and a new integrated foot care programme in the management of foot problems in JIA patients. The secondary aim of the study will be to determine what the optimum timing for intervention is in these patients by comparing the outcomes of pre-determined groups at different stages of the disease (children, adolescents and adults).

## Methods/design

### Study design and setting

**Figure 1:**
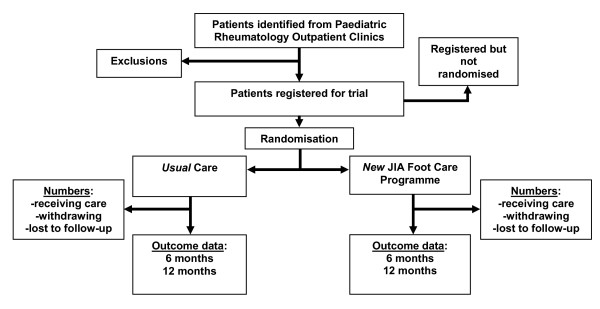
**Summary diagram of JIA Foot Care Programme trial design**.

FiJIA is a secondary-care based pragmatic exploratory randomised controlled trial where children (under 10 years of age) adolescents (between 10 and 16 years of age) and adults (16 years of age and over) will receive either standard care podiatry or intervention via a new foot care programme. A summary design is given in figure 1. A full economic evaluation will also be conducted.

### Ethical considerations

Full ethical approval for this study has been obtained (REC ref. 08/S0709/36). The trial steering committee will monitor the progress of the study at 6 month intervals.

### Identification of eligible patients

Children aged 16 and under will be recruited from the outpatient paediatric rheumatology clinic at The Royal Hospital for Sick Children, Yorkhill, Glasgow. Adults whose arthritis started in childhood will be recruited from the Centre for Rheumatic Diseases at Glasgow Royal Infirmary. Patients with JIA will be identified from existing clinical lists. At their routine clinical appointment, potential participants will be introduced to the study by their consultant and, if interested, the patients are to be introduced to the research team for more information. Patients will be screened for suitability according to the inclusion/exclusion criteria (see below). A minimum period of 24 hours will be provided before contacting potential participants by telephone to confirm willingness to participate in the trial and an appointment made for baseline visit.

### Inclusion/exclusion criteria

All participants must have JIA diagnosed by their consultant rheumatologist, according to the ILAR 2004 criteria. In addition, participants are to be included if they satisfy one of the following:

(i) Documented arthritis in the foot including small joints

(ii) Lower limb arthritis of two or more large joints (hips, knees, ankles and subtalar joints).

(iii) Widespread polyarthritis involving large and small joints.

Patients with only upper limb, jaw, or neck involvement only will be excluded, along with those unable to cooperate with the study. Participants may also be excluded from the study (prior to randomisation) on the basis that: -

(i) Any lesion detected during the ultrasound foot scan is not typical of synovitis (for example tumour, septic arthritis)

(ii) The lesion may require biopsy

(iii) The lesion requires referral for a second opinion

### Randomisation

Treatment allocation will be conducted by means of minimisation, a highly effective method of allocation which has been recommended for use in randomised controlled trials [33]. Using this method, treatment allocated to the next trial participant depends on the characteristics of those participants already enrolled, with the ultimate aim of 'minimising' the imbalance between groups [34]. Minimisation in this case will be adopted to achieve balance between groups using the following characteristics:

(i) Age

(ii) Gender

(iii) JAFI-IMP score

This method will be conducted by the same researcher (GH) using the minimisation software package Minim [35].

### Blinding

At baseline all assessors are blinded to the outcome of allocation. The standard care arm podiatrist will be blinded to participants' inclusion in the usual care arm of the trial to ensure that normal clinical practice remains so. The researcher conducting minimisation (GH) will be blinded to the results of all assessments and outcome measures except the JAFI-IMP score which is required to allocate participants through minimisation. The complex design of the trial necessitates that patients are aware of the outcome of allocation to either trial arm. However, in an attempt to reduce the placebo effect all patients will be informed that it is not known whether one intervention is more effective than the other. The primary outcome measure (JAFI) will be administered by one assessor (MB) who will remain blinded to the outcome of allocation at each time point.

### Initial assessments and outcome measures

At baseline all participants will be assessed to ensure they satisfy the inclusion criteria. Demographic and disease characteristic data such as age, gender, height, weight, disease sub-type, date of diagnosis, disease duration will also be recorded. All baseline assessments, primary and secondary outcome measures will be recorded prior to allocation to either the usual care arm or the intervention arm. The medical and physiotherapy care plans will be committed to paper prior to randomisation.

The primary outcome is the Juvenile Arthritis Foot Disability Index (JAFI) [31]. This questionnaire is organised by three dimensions related to impairment (9 items), activity limitation (14 items) and participation restriction (4 items) with a 5-point Likert scale for each item. Each point on the 5-point likert scale represents the frequency of the foot problem stated for that particular item during the past week (0 = Never, 1 = Occasionally/Less than once a week, 2 = Sometimes/Once a week, 3 = Frequently/Two to three times a week, and 4 = Always). Median scores are computed for each dimension. The JAFI is completed by parents of children <10 years and self-completed by adolescent children = 10 years of age. It has been shown to be to be valid and reliable for assessing foot-related impairment and disability among children/adolescents with JIA [31]. The JAFI has not yet been formally validated for use in adult patients with JIA. This questionnaire will be administered at baseline, then 6 (by post) and 12 months from baseline. The exploratory nature of the trial will permit in-depth examination of the suitability of the JAFI as a primary outcome measure for use in definitive multi-centre trials.

Diagnostic US will be undertaken using established methodologies for the foot and ankle employing B-mode and colour and Power Doppler [36-38]. Accessible aspects of first through fifth metatarsophalangeal (MTP) joints will be scanned to include the dorsal and medial 1^st ^MTP joint; dorsal and plantar 2^nd^-4^th ^MTP joints and the dorsal, lateral and plantar aspect of the 5^th ^MTP joint [37]. Proximally, the tibio-talar, subtalar, talonavicular, calcaneocuboid, cuneonavicular, and tarsometatarsal joints will be scanned in both longitudinal and transverse planes, along with the calcaneal and retrocalcaneal bursa, the plantar fascial calcaneal enthesis, the peroneal tendons, tibialis anterior, the Achilles tendon, tibialis posterior, flexor hallucis longus and flexor digitorum longus [36,38]. The semi quantitative grading system (see table 1) by Szkudlarek et al [37] will be modified used and used to score the presence or absence of US detected pathology. Changes in US derived pathology will be recorded between baseline and 12 months. These scores will be site and tissue specific (joint, tendon, bursa) rather than summated to capture response to localised treatment, for example, tibialis posterior synovitis.

**Table 1:** Semi-quantitative ultrasound scoring system

**Pathological feature**	**Definition**	**Scoring**
Joint Effusions	Defined as a compressible anechoic intra-capsular area	0- no effusion
		
		1- minimal amount of effusion
		
		2- moderate amount of effusion without distension of the capsule
		
		3- Extensive amount of effusion with distension of the joint capsule

Synovitis	Defined as a non-compressible hypoechoic intra-capsular area (synovial thickening)	0- no synovial thickening
		
		1- minimal synovial thickening (filling the angle between adjacent bones without bulging over the line linking the tops of the bones)
		
		2- synovial thickening bulging over the line of the tops of the peri-articular bones but without extension along the bones diaphysis
		
		3- Synovial thickening bulging over the lines linking the tops of the peri-articular bones and with extension to at least one diaphysis

Bone erosions	Changes in the bone surface of the area adjacent to the joint	0- Regular bone surface
		
		1- Irregularity of the bone surface without formation of a defect in the surface of the bone seen in 2 planes
		
		2- Defect in the surface of the bone seen in 2 planes

		3- Bone defect creating extensive bone destruction

Power Doppler	Display of signal flow in the synovium	0- No flow
		
		1- Single vessel signals
		
		2- Confluent vessel signals in less that half of the synovium
		
		3- Vessel signals in more than half of the area

To objectively measure lower limb and foot function, spatiotemporal gait parameters (walking speed, double-support time and symmetry index) will be assessed using an instrumented walkway (GaitRITE, CIR systems, Clifton, NJ, USA). Plantar pressure and force distribution will be measured using a pressure platform (Emed NT, Novel GmbH, Munich, Germany) using standardised protocols as previously developed in adult rheumatoid arthritis and JIA research [27,39,40]. From the gait analysis, six key variables will be selected: peak pressure in the forefoot; contact area in the toes; contact area in the midfoot; transfer of centre of pressure through the foot, and the peak ground reaction force during the loading response and terminal stance phases. Walking speed (m/s), stride length (m), cadences (steps/min), cycle time (% gait cycle), and double support time will be selected as a core set of spatiotemporal gait parameters and compared against age/gender normative values [27,39].

Arthritis activity measures will be assessed using the Core Outcome Variables for JIA, a validated clinical assessment for disease activity which correlates with adult measures. JIA patients under 16 years will have the Core Outcome Variables recorded (consisting of the CHAQ questionnaire completed by the patient or parent, active and limited joints, and physician global assessment completed by the doctor in clinic). For adults with JIA these will be the physician global assessment, pain assessment, number of tender, swollen and deformed joints and duration of early morning stiffness and HAQ. These adult and paediatric measures are comparable. The Glasgow Enthesitis Score, a non-validated measure of enthesitis severity, will be recorded by the examining doctor in all patients.

A variety of secondary outcomes will be recorded at baseline and at the final study visit (12 months from baseline). The Childhood Health Assessment Questionnaire (CHAQ) and the Health Assessment Questionnaire (HAQ) will be used as measures of overall function/disability in children/adolescents and adults respectively [41,42], while the EQ5D will be employed to measure general HRQoL, and will be used specifically within the economic evaluation [43].

Patients will undergo an examination of their foot and ankle joints to assess for active synovitis or limited joint ranges of motion. The presence or absence of these clinical features will be summated to derive active and limited foot joint counts ranging 0–28. Foot deformity will be assessed using the Structural Index (SI) to provide summated scores for rearfoot/ankle deformity (0–14) and forefoot deformity (0–24) [44]. The SI is a validated semi-quantitative scoring mechanism for adult subjects with Rheumatoid Arthritis [44]. It has good face validity for use in subjects with JIA [16] but it has not yet been formally validated for use within this patient group.

### Interventions

#### Usual care

Participants randomised to usual care (standard podiatry) will receive normal outpatient medical care. Those in current receipt of foot care via (either adult or paediatric) podiatry services will continue to receive care, while new referrals will also be permitted. Referrals will be monitored to ensure that medical staff referral patterns do not change once the study has started.

#### Intervention

**Figure 2:**
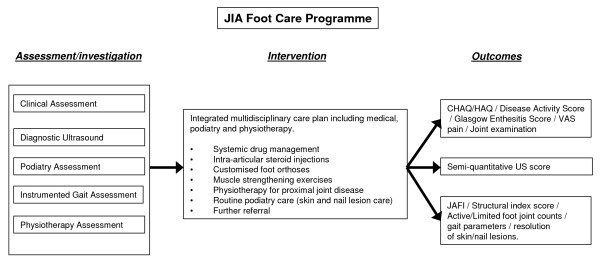
**Summary diagram of JIA Foot Care Programme (assessment/intervention/outcome)**.

Following US identification of the anatomical structures involved by inflammatory lesions, and altered foot and lower limb function by instrumented gait analysis, a plan of appropriate clinical action will be taken by the (paediatric and adult) rheumatologists (JGM/RS), the podiatrists (JW, DET & GH) and physiotherapist (LF) (summary design is given in figure 2). If intra-articular joint injections are to be prescribed by the rheumatologists (JGM/RS), then these will be conducted using ultrasound guidance within 1 month of initial consultation. The care plans will be agreed through discussion between the multi-disciplinary team based on their interpretation of the initial assessments. Participants will receive individualised care packages comprising combinations of foot orthoses and footwear treatments, physical therapies including stretching and muscle strengthening and standard podiatry care for problems such as skin callus and in-growing toenail; these will be delivered on the same day where possible. Customised orthoses will be manufactured via an external laboratory (Firefly Orthoses, Sligo, Ireland) according to the standardised order form, and will be ready for fitting within two weeks. As part of the new programme, rapid podiatry access will also be provided for unscheduled care episodes such as skin and soft-tissue foot infections, predominantly in-growing toenail, associated with disease modifying therapies. Finally, further multidisciplinary care will be used to include orthotist services when non-standard ankle/foot orthoses are indicated. Clinicians will use a core set of outcomes (JAFI, disease activity score, gait parameters and US) to chart progress and to modify individual treatment programmes during the trial period.

#### Patient follow up procedures

All participants regardless of allocation will undergo another full assessment once they have proceeded to the end of the trial (12 months from baseline). In the interim period, the primary outcome questionnaire (JAFI) will be posted at 6 months from baseline with a stamped-addressed envelope in order to complete and return to the study personnel. Non-responders will be sent a second questionnaire within 2 weeks, followed by a third a further two weeks later, and then contacted by written letter/telephone. Participants in the intervention arm will receive return appointments every 3 months. Any additional appointments within the intervention arm will be made within the scope of normal clinical practice.

#### Economic evaluation

An economic evaluation aims to assess whether an intervention provides value-for-money relative to its comparator. There are three approaches which can be employed to assess this: cost-effectiveness analysis (CEA), cost utility analysis (CUA) and cost benefit analysis (CBA). This trial provides a unique opportunity to not only assess value-for-money but also consider the issue of outcome measurement in children and adults where parents are used as a proxy respondent, and to further explore the aspects surrounding parental and patient preferences for health care. Therefore, a relatively exhaustive evaluation is proposed which will utilise all three of the economic evaluation approaches.

### Cost effectiveness analysis

As described above, at baseline JAFI will be recorded. Health care resource use will be collected from patient notes and parents/adult suffers will also provide details of other health care use (primary care use), out-of-pocket expenses and productivity loss using a self-completing questionnaire. Health care resource use will be valued using national published costs [45-47] while lost productivity will be valued using the human capital approach.

At 6 and 12 months from baseline outcomes will be reassessed. The difference in outcome between baseline and 6 and 12 months will provide a measure of effectiveness. Costs incurred during the trial will be aggregated to represent an annual cost, and both direct health care costs and indirect/societal costs of the intervention and standard care will be calculated. The economic analysis will compare the costs and outcomes in each arm, and it will estimate the (additional) cost per (additional) unit of improvement of the JAFI, which will be presented in the form of an incremental cost effectiveness ratio (ICER). Confidence limits for the ICER will be estimated using bootstrapping, and the analysis will also produce cost effectiveness acceptability curves (CEACs), which provide a presentation of uncertainty in terms of the threshold of acceptability.

### Cost utility analysis

A CUA will be conducted similarly to that described above, whereby the EQ5D will be completed at baseline, and at 6 months and 12 months. The UK tariff [48] will be used to value the health states and QALYs will be estimated using the area under the curve method [49]. An ICER using the cost per QALY gained will be estimated, and in this instance can be more informative than cost per improvement in JAFI, as should the intervention prove to be more effective but also more costly, then it is possible to compare the cost per QALY gained with the National Institute for Health and Clinical Excellence (NICE) threshold.

Notably the EQ5D has only been validated for use in adults, and while there is a child version available, it has not yet been validated. In order to explore the issue of child responses versus parent proxies, given that the target population includes children as well as adults who developed JIA as a child, it is proposed that the children in the study will complete the child version of the EQ5D, while their parents will complete the adult version on behalf of their children. The resulting scores will be compared, and will be used to inform a sensitivity analysis of the CUA to determine whether they influence any decision on value-for-money.

### Cost benefit analysis

There are a number of techniques available with which to elicit a monetary value of the benefit of health care. Discrete choice experiments (DCE), more formally known as conjoint analysis, are a commonly employed approach to eliciting stated preferences [50]. When DCEs include cost information, it is possible to manipulate the results to given information on respondents' willingness-to-pay (WTP) for a service, such that a CBA can be undertaken.

A literature review will be conducted to determine health and non-health preferences (attributes) for service provision, this together with semi-structured interviews and focus group discussions with patients, parents and health professionals, will inform the development of the attributes and levels for the DCEs [51-53]. Once the attributes have been identified, and the levels and costs set by the project team, an orthogonal design will be created. Both the control and the treatment arm groups will complete the DCE questionnaire (parents in children under 16 years of age) at baseline. This will provide information from which to indirectly establish willingness-to-pay, and undertake a CBA. As an added dimension it is proposed that patients/parents will undergo the same experiment at the completion of the trial. This will allow an evaluation of whether exposure to the intervention (as for those in the intervention arm) changes their preferences for the intervention.

### Sample size

The sample size for the study has been pre-determined by power analysis of the primary outcome measure; the impairment domain of the JAFI questionnaire (JAFI-IMP. Sixty patients (30 in each trial arm) will allow detection of a 1 point reduction in the JAFI-IMP score (a conservative assumption determined by the trial steering committee) at six months from baseline at a significance level of p < 0.05 and power of 90%. These figures take into account the estimated potential participant drop out rate over the first six months of the trial (8 patients in each trial arm). The exploratory nature of the trial will permit in-depth examination of the suitability of the JAFI as a primary outcome measure for use in definitive multi-centre trials.

### Statistical analysis

The primary analysis will compare the JAFI scores for each dimension between the intervention and control group at six months from baseline. This is to be conducted using a two-sided t-test. Should the distribution of change scores be skewed, a Mann Whitney test may be used to analyse the primary outcome variable in preference to a t-test. The longitudinal JAFI scores, at 0, 6, and 12 months will be analysed using repeated measures ANOVA with post hoc testing to identify significant differences between treatment groups and time points.

Core outcome variables scores for each group will be compared using a confidence interval for the difference in proportions. This will be based on a normal approximation to the binomial distribution.

Changes in predetermined gait variables (see Patients assessments and outcome measures section) from baseline to 12 months from baseline will be investigated within each treatment group using a one-sample procedure (t test or Signed Ranks test) and compared across treatment groups using the equivalent two-sided procedure (t test or Mann-Whitney test).

## Competing interests

The authors declare that they have no competing interests.

## Authors' contributions

JW, JGM, DT, RS, GW and GH identified the research question and obtained funding for the study. JW, JGM, DT, RS, PL, JM, LF, MB and GH all contributed to aspects of the study protocol and design. PL played a leading role in the development of the health economic evaluation. JW and GH drafted this paper and all authors have read and approved the final version.
